# Direct exciton emission from atomically thin transition metal dichalcogenide heterostructures near the lifetime limit

**DOI:** 10.1038/s41598-017-09739-4

**Published:** 2017-09-28

**Authors:** Jakob Wierzbowski, Julian Klein, Florian Sigger, Christian Straubinger, Malte Kremser, Takashi Taniguchi, Kenji Watanabe, Ursula Wurstbauer, Alexander W. Holleitner, Michael Kaniber, Kai Müller, Jonathan J. Finley

**Affiliations:** 1 Walter Schottky Institut and Physik Department, Technische Universität München, Am Coulombwall 4, 85748 Garching, Germany; 2 Nanosystems Initiative Munich (NIM), Schellingstr, 4, 80799 Munich, Germany; 3 National Institute for Materials Science, Tsukuba, Ibaraki 305-0044 Japan

## Abstract

We demonstrate the reduction of the inhomogeneous linewidth of the free excitons in atomically thin transition metal dichalcogenides (TMDCs) MoSe_2_, WSe_2_ and MoS_2_ by encapsulation within few nanometre thick hBN. Encapsulation is shown to result in a significant reduction of the 10 K excitonic linewidths down to ∼3.5 meV for n-MoSe_2_, ∼5.0  meV for p-WSe_2_ and ∼4.8 meV for n-MoS_2_. Evidence is obtained that the hBN environment effectively lowers the Fermi level since the relative spectral weight shifts towards the neutral exciton emission in n-doped TMDCs and towards charged exciton emission in p-doped TMDCs. Moreover, we find that fully encapsulated MoS_2_ shows resolvable exciton and trion emission even after high power density excitation in contrast to non-encapsulated materials. Our findings suggest that encapsulation of mechanically exfoliated few-monolayer TMDCs within nanometre thick hBN dramatically enhances optical quality, producing ultra-narrow linewidths that approach the homogeneous limit.

## Introduction

In the group of atomically thin two-dimensional (2D) materials the transition metal dichalcogenides MoS_2_, MoSe_2_, WS_2_ and WSe_2_ reveal fascinating photophysical properties owing to their direct gap and strong light-matter interactions^1,2^. The weak dielectric screening results in emission dominated by excitonic processes, with exciton binding energies on the order of several hundred meV^3,4^ that follow a non-hydrogenic Rydberg series^5^. However, in the vast majority of reports to date the linewidths of the free excitons exhibit significant inhomogeneous broadening. This is typically attributed to the local spatial inhomogeneity of the substrate, adsorbed atoms and molecules on the surface due to the large surface-to-volume ratio and different doping and dielectric screening conditions that are highly sensitive to the choice of substrate. Broad linewidths of the exciton emission of ∼50 meV for MoS_2_^6^, ∼40–75 meV for WS_2_^7,8^, ∼5 meV for MoSe_2_^9^ and ∼10 meV for WSe_2_^10^ have been reported in photoluminescence experiments(PL), while time-domain spectroscopy^11–13^ and recent theory^14^ report homogeneously broadened luminescence linewidths in the range of ∼2–6 meV depending on the material system. The healing of sulphur defects using sulphuric superacids increases the optical quantum yield and reduces the linewidths at room temperature^15–17^ from ∼70 meV to ∼55 meV. However, low temperature studies of treated MoS_2_ monolayers^17^ show that the linewidths still remain in the order of ∼15 meV. Very recently, it has been shown that MoS_2_ is particularly sensitive to photo-induced irreversible changes resulting in broad luminescence from overlapping neutral and charged exciton emission^18^. Measurements performed using ultra-low excitation power densities reveal distinct peaks for neutral and charged excitons with linewidths of ∼15 meV for MoS_2_ similar to Se-based TMDCs^18^.

In this letter, we present an optical study of TMDCs encapsulated within hBN and demonstrate that encapsulation leads to a significant reduction of the linewidth observed in photoluminescence (PL) experiments, towards the radiative limit. We systematically probe modifications in the luminescence linewidth after each stacking step and extract key parameters such as the exciton peak position, relative intensities of exciton and trion recombination and peak linewidths. We also show that annealing of the heterostructure improves the spatial homogeneity of the TMDC and, thus, of the observed luminescence. From our results, we make three major observations upon hBN encapsulation: (i) the linewidths of free excitons are significantly reduced down to a few meV approaching the homogeneous linewidth limit, (ii) the surface is protected, preventing samples against irreversible photo-induced spectral changes and (iii) encapsulation effectively lowers the Fermi level, reducing emission from negatively charged excitons in MoSe_2_, while increasing the emission from positively charged excitons in WSe_2_ due to protection against physisorption and impurities from the substrate.

## Results and Discussion

### Photoluminescence of encapsulated MoSe_2_

**Figure 1:**
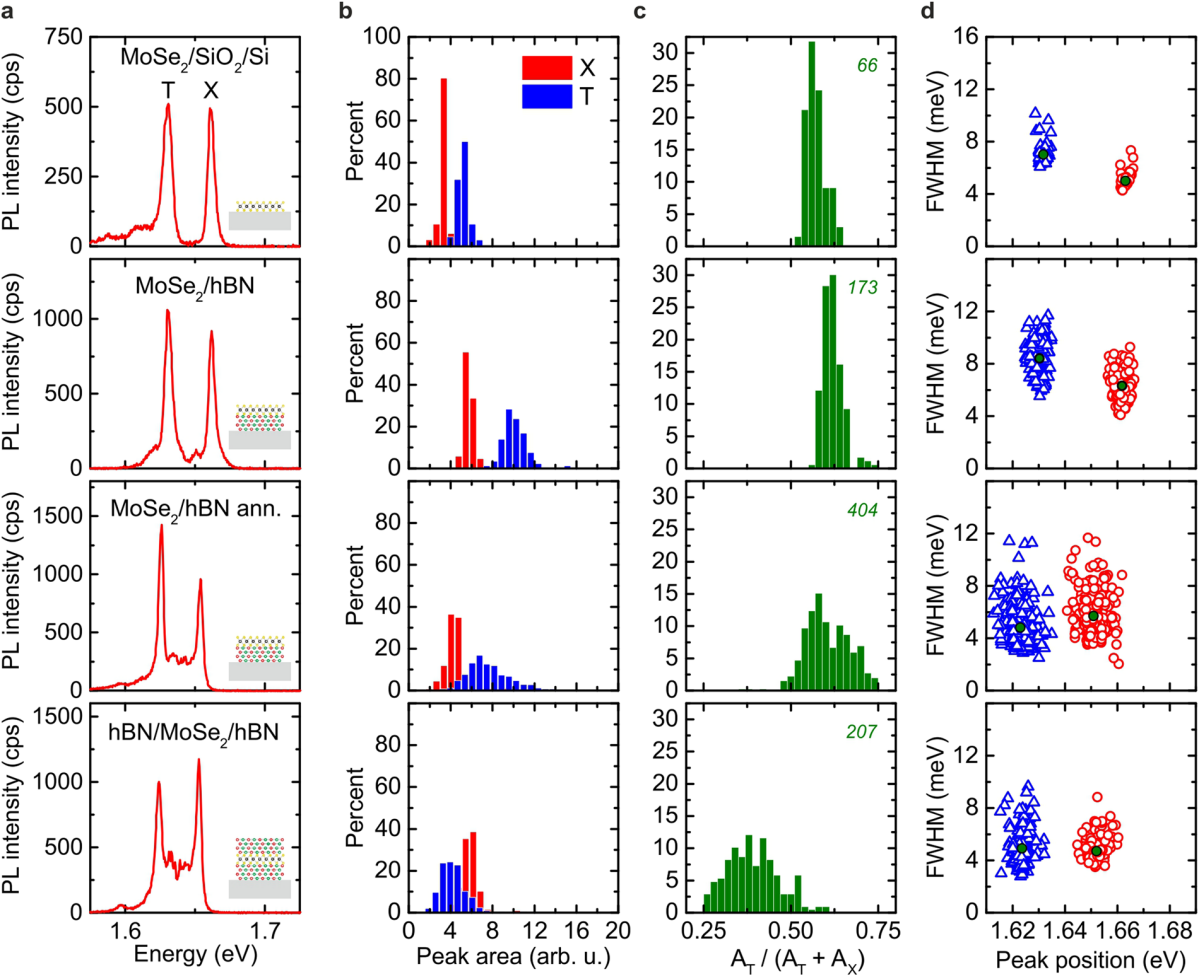
MoSe_2_ photoluminescence spectra and statistics. (**a**) Typical low-temperature (10 K) μ-PL spectra of MoSe_2_ on SiO_2_, MoSe_2_ on hBN, MoSe_2_ on hBN after annealing and MoSe_2_ encapsulated between hBN. Emission is observed from the neutral (X) and charged exciton (T) transitions. (**b**) Histogram of the peak areas of X (red, *A*_*X*_) and T (blue, *A*_*T*_). (**c**) Corresponding relative spectral weight $R={A}_{T}/({A}_{T}+{A}_{X})$. The green italic number represents the number of fitted spectra used for the histograms. (**d**) Correlated distribution of Lorentzian linewidths and corresponding peak positions of X (red circles) and charged (blue triangles) exciton. The green circles and triangles denote the corresponding mean values.

To probe the impact of the proximal substrate and explore the benefits of hBN encapsulation, we performed spatially resolved PL measurements and statistically analyse the emission spectra at different positions on the sample surface. Note, in our analysis we disregard spectra recorded from the edge of the flake and obviously damaged parts of the sample, as identified by conventional light microscopy. From the measurements, we extract the peak positions, full widths at half maximum linewidths (FWHM) and relative intensities of the neutral exciton (X) and charged trion (T) by fitting with Lorentzian peaks. Figure 1 compares examples of spectra of MoSe_2_ on SiO_2_, MoSe_2_ on hBN, MoSe_2_ on hBN after annealing and MoSe_2_ sandwiched between hBN and after annealing. The corresponding statistical analysis of peak position, exciton linewidth and peak area for the different MoSe_2_/substrate configurations are shown in Fig. 1b–d, respectively. Note that in order to obtain the best comparison, in the case of MoSe_2_ on hBN we scan the same area after subsequent steps of stacking and annealing to trace the impact of the encapsulation on the spectral evolution. A typical spectrum recorded from MoSe_2_ on SiO_2_ is presented in Fig. 1a (top). It exhibits pronounced emission from trions, typically attributed to extrinsic effects such as doping from the substrate, mediated through trap states^6,9,10,19^ and intrinsic doping resulting from chalcogen vacancies and adsorbates that are reported to occur in mechanically exfoliated flakes^20,21^. We obtain a qualitative measure of the doping by analysing the areas of the neutral and charged exciton *A*_*X*_ and *A*_*T*_ and their relative spectral weight *R *= *A*_*T*_/(*A*_*T*_ + *A*_*X*_). Figure 1b shows the peak areas, while the corresponding relative spectral weights are presented in Fig. 1c. The emission intensity for MoSe_2_ on SiO_2_ is higher for trions than for neutral excitons which is reflected by values of R > 0.5 in Fig. 1c. This remains unchanged when MoSe_2_ is stacked on top of ∼14 nm thick hBN, and also after annealing that only results in a slightly decreased total peak area. However, fully encapsulated MoSe_2_ exhibits a higher X peak-area compared to T with R < 0.5. This behaviour is indicative of an effective lowering of the Fermi level in the crystal inhibiting trion formation. We attribute this to protection against surface charge contributions from the SiO_2_ layer on the substrate material^6^, as hBN naturally shows low defect densities over large areas^22^. This effect is strongest in the fully encapsulated configuration. Since the MoSe_2_ is exposed to ambient conditions during and after fabrication in the previous configurations, the TMDC surfaces are free to physisorption of ambient molecules^23,24^, most likely H_2_O due to its polarity. Thus, we assume that the impact of the hBN substrate is reduced due to frozen adsorbates possibly at defects such as selenium vacancies on the TMDC surface.

Changes in the dielectric environment and doping can further influence the exciton peak positions and the linewidths (Fig. 1d) ^25^. Here, we directly correlate peak positions and linewidths. The exfoliated MoSe_2_ on SiO_2_ shows exciton peak positions of *P*_*X*_ = (1663.1 ± 1.2) meV and *P*_*T*_ = (1631.8 ± 1.3) meV with a binding energy of *E*_*T*_ ∼ 31 meV which is typically observed in literature^9,26^. Stacking MoSe_2_ on hBN results only in a slight redshift by *ΔE* ∼ −2 meV and a slightly broader distribution as can be seen in Fig. 1b. This redshift is consistent with recent calculations^25^ and measurements^27^ considering the change in the refractive index of the substrate from ${{n}}_{{{SiO}}_{{2}}}=1.457$ to *n*_*hBN*_ = 2.2 at the neutral exciton resonance^28,29^.

Annealing results in an additional redshift, and a total shift to a lower energy by Δ*E* ∼ −12 meV compared to pristine MoSe_2_. This is accompanied by a much broader distribution of peak positions. The sandwiched and annealed MoSe_2_ structure exhibits the strongest redshift of Δ*E* ∼ −12 meV. Yet, the statistical spread of the peak position distribution is significantly reduced from *s*_*X*_ = (6.8  ±  0.1) meV to *s*_*X*_ = (2.8  ±  0.1) meV (see Supplementary Fig. S2), as depicted in the bottom panel in Fig. 1d. Moreover, the trion binding energy decreases from *E*_*X*_ − *E*_*T*_ = (31 ± 3) meV to (28 ± 3) meV after the annealing step possibly resulting from the modification of the dielectric environment and a change in extrinsic doping^25^. In general, we observe that the symmetric dielectric hBN environment of the MoSe_2_ flake combined with annealing results in the sharpest distribution of emission energies, indicative of the highest homogeneity within the MoSe_2_ flake. The statistical analysis of the linewidths for MoSe_2_ on SiO_2_ reveals average values of *w*_*X*_ = (5.0 ± 0.5) meV and *w*_*T*_ = (7.0 ± 0.8) meV for X and T excitons, respectively. Stacking MoSe_2_ on hBN results in significantly higher linewidths of *w*_*X*_ = (6.3 ± 1.0) meV and *w*_*T*_ = (8.4 ± 1.3) meV with a much broader variation in obtained values. Annealing reduces the linewidth to *w*_*X*_ = (5.7 ± 1.5) meV and *w*_*T*_ = (4.8 ± 1.5) meV while capping with hBN further reduces the X linewidth to *w*_*X*_ = (4.7 ± 0.9) meV, keeping the T linewidth at *w*_*T*_ = (4.9 ± 1.3) meV. Interestingly, annealing reveals much higher variance of values which is significantly narrowed upon capping. However, for investigating the linewidths not only the average values are important but also the lowest values obtained. Importantly, for MoSe_2_ encapsulated in hBN we observe values as low as *w*_*X*_ ∼ 3.5 meV, almost reaching the homogeneous linewidths recently reported in time-resolved four-wave-mixing experiments^12,13^ and theoretical calculations^14^ of *w*_*X*_ ∼ 2.1 meV, *w*_*X*_ ∼ 3.4 meV and *w*_*X*_ ~ 5.5 meV, for lattice temperatures of *T* = 6.5 K and 10 K, respectively.

With the dependence *γ*_*rad*_ ∝ 1/*n*_Substrate_ for the radiative linewidth broadening^14,30^, changing the substrate material reduces *γ*_*rad*_ by a factor of ${n}_{{{\rm{S}}{\rm{i}}{\rm{O}}}_{2}}$/*n*_hBN_ ≈ 0.66. This then produces a radiative rate which would be quantitatively consistent with the narrowest linewidths measured in our study. Beside radiative broadening, primarily exciton-phonon coupling has been identified as broadening mechanism^14^. Moreover, we attribute the observed remaining broadening of the linewidth to spatial inhomogeneities of the TMDC as a result of the exfoliation procedure and residual polymer bubbles between the interfaces of the monolayer crystal and the surrounding hBN.

### Photoluminescence of encapsulated WSe_2_

**Figure 2:**
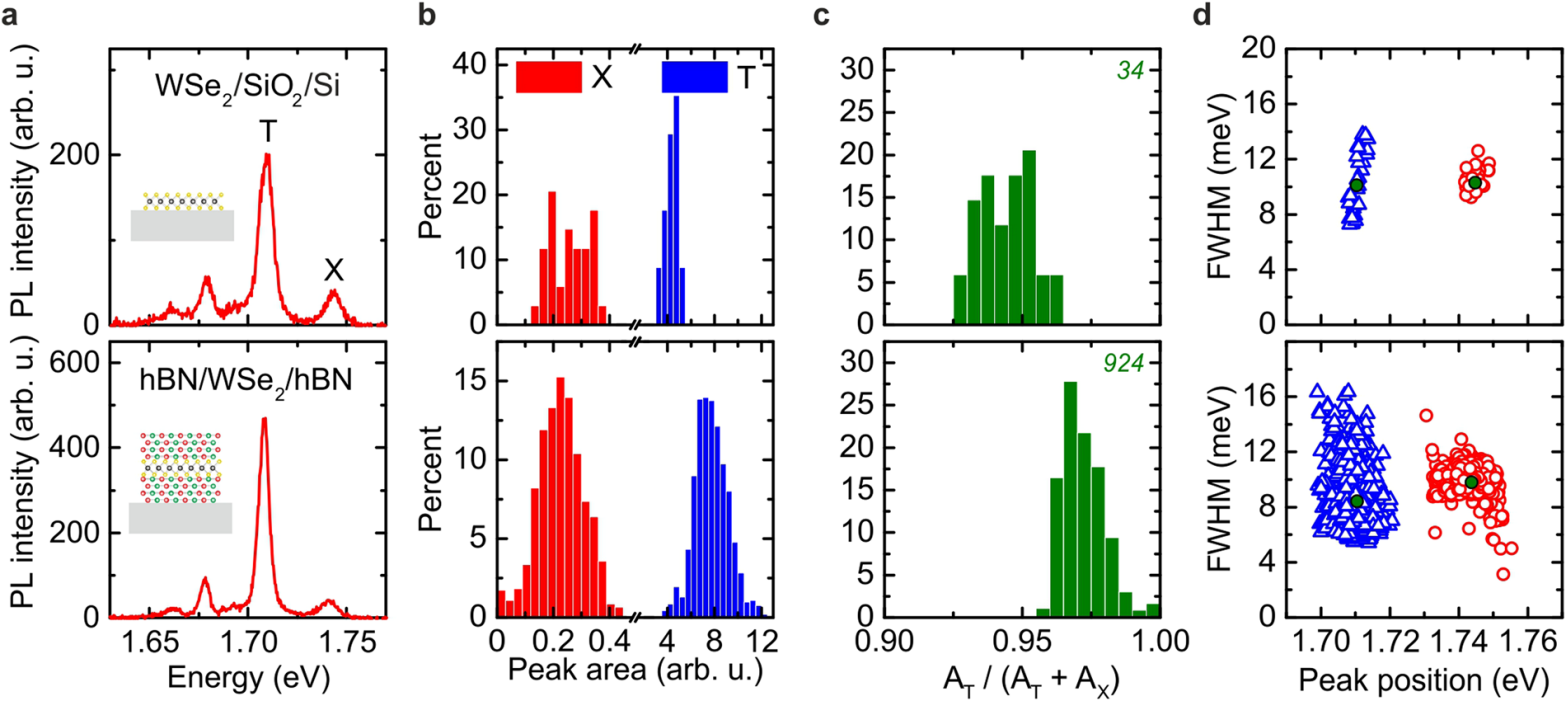
WSe_2_ photoluminescence spectra and statistics. (**a**) Typical low-temperature (10 K) μ-PL spectrum WSe_2_ on SiO_2_ and encapsulated within hBN featuring emission from the neutral (X) and charged exciton (T). (**b**) Histogram of peak areas of X (red, *A*_*X*_) and T (blue, *A*_*T*_). (**c**) Corresponding relative spectral weight *R* = *A*_*T*_/(*A*_*T*_ + *A*_*X*_). The green italic number represents the fitted spectra used for the histograms. (**d**) Correlated distribution of Lorentzian linewidths and corresponding peak positions of X (red circles) and charged (blue triangles) exciton. The green circles and triangles denote the corresponding mean values.

We repeated the fabrication scheme and optical experiments discussed above for MoSe_2_ with WSe_2_. Since we found the most significant improvement in optical quality for TMDCs that are fully encapsulated in hBN, we compare only the two cases of WSe_2_ on SiO_2_ and WSe_2_ encapsulated in hBN after annealing. Typical spectra for WSe_2_ on hBN are presented in Fig. 2a. The relative spectral weights *R* are shown in Fig. 2b. Comparing the relative peak areas of the neutral and charged excitons, results in a trend opposite to that for MoSe_2_. For WSe_2_, the relative intensity of the charged exciton increases by a factor of two upon encapsulation with hBN. We explain this trend by the difference in intrinsic doping of TMDCs present in our experiments. The MoSe_2_ crystal employed in this work is n-doped, consistent with measurements on electrically contacted monolayer devices (see Supplementary Section S1), which results in a negatively charged exciton. In contrast, the WSe_2_ is p-doped, resulting in emission from positively charged excitons (see Supplementary Fig. S3). Thus, the hBN encapsulation enables a higher positively charged exciton formation rate.

Upon encapsulation, we observe a reduction of the neutral exciton emission linewidth from (10.3 ± 0.7) meV to (9.8 ± 1.4) meV, whilst the trion emission linewidth reduces from (10.1 ± 2.1) meV to (8.4 ± 1.9) meV. However, this effect is accompanied by a higher overall spread in the linewidth distribution for the capped material. A similar trend is observed for the distribution of peak positions. However, here only a slight redshift is observed. Notably, we observe linewidths as low as *w*_*X*_ ∼ 5 meV for the neutral exciton and *w*_*T*_ ∼ 5.5 meV. Recent four-wave-mixing measurements^11,13^ and theoretical work^14^ report and predict homogeneous linewidths of *w*_*X*_ ∼ 6.1 meV, *w*_*X*_ ∼ 4.7 meV and *w*_*X*_ ∼ 6.5meV, respectively.

Optimised stacking processes, reducing bubble formation and wrinkling of the 2D materials could lead to desired purely lifetime broadened emission of the TMDCs.

### Photoluminescence of encapsulated MoS_2_

**Figure 3:**
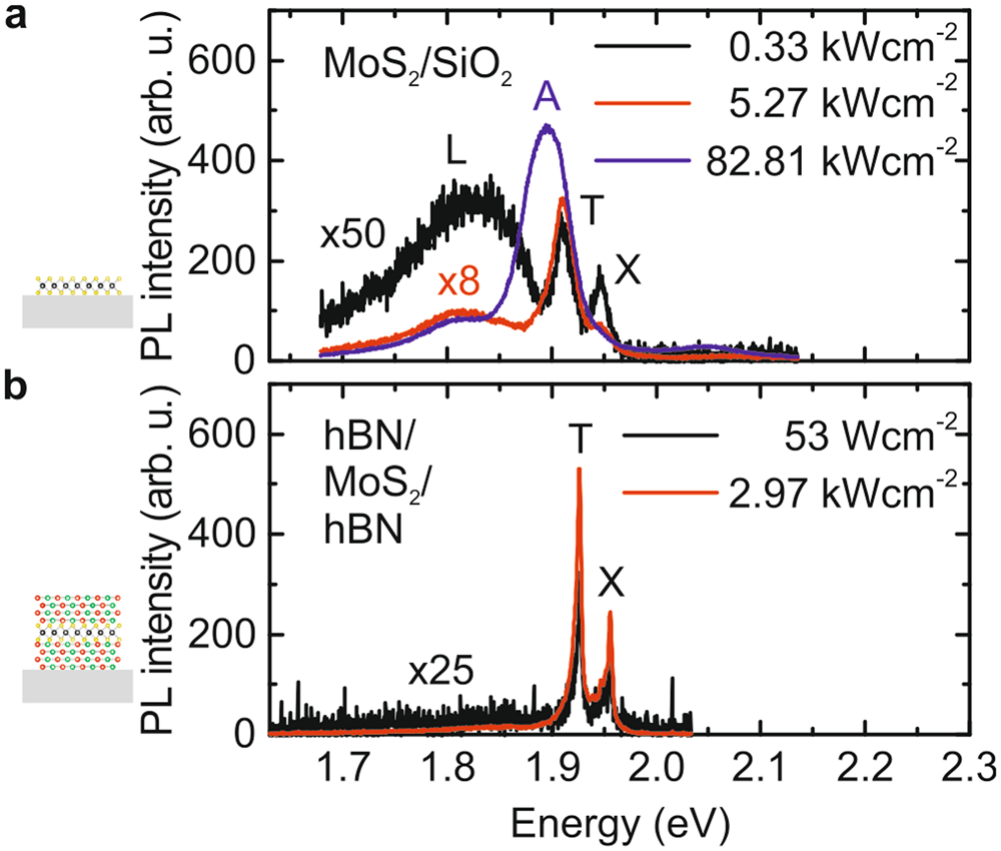
Power dependent MoS_2_ photoluminescence spectra. (**a**) Typical μ-PL spectrum of MoS_2_ on SiO_2_ for a low (black) moderate (red) and high (blue) excitation power featuring the A-peak (blue spectrum) neutral and charged exciton emission and the L-peak at lower energies. (**b**) Typical μ -PL spectrum of hBN encapsulated MoS_2_ for a low (black) and high (red) excitation power reveals sharp neutral and charged exciton emission and no emission from the L-peak.

In addition to the Se based TMDCs, we also applied our encapsulation scheme to MoS_2_ which in past experiments showed comparatively broad emission from the A-exciton^1,2,6^. This is attributed to inhomogeneous broadening of the emission from neutral and charged excitons that is typically so large that the two peaks are not resolved. Typical PL from MoS_2_ exfoliated on SiO_2_ is presented in Fig. 3a. For very low excitation power densities of 0.33 kWcm^−2^, the spectrum (black curve) reveals emission from the neutral exciton X at (1947.4 ± 0.3) meV, charged excitons T at (1910.7 ± 0.3) meV and pronounced emission from the low energy L-peak is observed located ∼100 meV below X. This broad emission is attributed to defect-related exciton emission^1,2,31^. Upon increasing the excitation power density to 5.27 kWcm^−2^ (red curve in Fig. 3a) the emission from the neutral exciton vanishes while charged exciton emission dominates. Meanwhile the emission from the L-peak saturates, and its contribution reduces compared to the charged exciton emission. When further increasing the excitation power density to values as high as 83 kWcm^−2^ (blue curve in Fig. 3a), the emission merges to the broad A-exciton peak normally observed in luminescence studies of MoS_2_ with a linewidth of *w*_*A*_ ∼ (53.6 ± 0.8) meV^2^. Note that these photo-induced changes in the form of the PL spectrum in our studies were found to be irreversible, consistent with recent findings^18^. For the lowest excitation power densities investigated, the neutral and charged excitons exhibit linewidths of *w*_*X*_ ∼ (14.7 ± 0.7) meV and *w*_*T*_ ∼ (23.4 ± 0.8) meV. Here, a full statistical analysis was not possible due to the photo-induced changes in the optical spectra. In strong contrast, encapsulation of MoS_2_ and annealing significantly enhances the optical emission properties. The PL (Fig. 3b) exhibits bright emission from free excitons. The neutral exciton at (1955.8 ± 0.5) meV and the trion emission at (1926.2 ± 0.5) meV is now blue shifted by (8.4 ± 1.0) meV and (15.5 ± 1.0) meV compared to the MoS_2_ on SiO_2_ configuration, respectively. By comparing the bare monolayer on SiO_2_ at ∼5 kWcm^−2^ to the encapsulated MoS_2_ at ∼3 kWcm^−2^ (red curves in Fig. 3a and b), we observe that the relative spectral weight strongly shifts from ∼ 0.94 towards lower values of ∼ 0.75. This behaviour of the relative spectral weight of the charged trion emission indicates an effectively lowered Fermi level in the MoS_2_. The overall blueshift is accompanied by a strong decrease in X and T linewidths down to *w*_*X*_ ∼ (4.8 ± 1.0) meV and *w*_*T*_ ∼ (6.8 ± 0.9) meV, consistent with recent work by Dey *et al*.^13^ reporting a homogeneous linewidth of *w*_*X*_ ∼ 6.6 meV in time-resolved four-wave-mixing measurements.

Importantly, we observe no emission from the L-peak indicative of defects and adsorbates^1,2,31^ for the fully encapsulated sample. Such features are observed for all other sample configurations, further highlighting the importance of surface protection. Furthermore, both exciton species are well resolved and we observe no photo-induced changes even for the highest excitation power (83 kWcm^−2^) used in our experiments.

## Conclusion

In summary, we have investigated the impact of hBN encapsulation on the optical properties of several TMDCs through statistically analysing low temperature photoluminescence experiments. Encapsulation distinctly reduces exciton linewidths and further protects the TMDCs against unwanted doping contributions from substrates or ambient molecules. Moreover, surface protection especially enhances the optical quality of MoS_2_, resulting in very clean spectra and revealing sharp emission from neutral and charged exciton without the presence of any irreversible photo-induced changes. Our findings suggest that encapsulation of TMDCs is essential for accessing the interesting photophysical properties of MoS_2_ and enables more sophisticated future optoelectronic devices.

During the writing of this manuscript we recognised related work reported by Cadiz *et al*.^32^ and Ajayi *et al*.^33^.

## Methods

### Sample preparation

The monolayer TMDCs studied in this letter are mechanically exfoliated onto degenerately n-doped Si substrates covered with a 285nm thick layer of wet-thermally grown SiO_2_. The heterostructures are stacked using the dry viscoelastic transfer method^34^, whereby we iteratively stacked hBN/TMDC/hBN onto the Si/SiO_2_ substrate. The hBN layer thicknesses range from 10nm to 70nm (AFM measurements). After stacking, the heterostructures were annealed at 150 K for 20 min to remove water and polymer accumulated into bubbles and improve the sample homogeneity (see Supplementary Fig. S1).

### μ-PL measurements

All photoluminescence (PL) experiments were performed using a confocal microscope at 10 K. The continuous-wave excitation energy was kept at 2.33 eV (Nd:YAG) and an excitation power density of 0.66 kWcm^−2^, unless otherwise noted. The spatial mode field diameter of the focal spot (1/*e* ^2^ contour) was ∼1.1 μm. The detected light was filtered with a steep fluorescence filter with a transmission cut-on energy 11.7 meV below the laser excitation energy.

The datasets generated during and/or analysed during the current study are available from the corresponding author on reasonable request.

## Electronic supplementary material

Supplementary information

## References

[1] Splendiani A (2010). Emerging photoluminescence in monolayer MoS_2_. Nano Letters.

[2] Mak KF, Lee C, Hone J, Shan J, Heinz TF (2010). Atomically Thin MoS_2_: A New Direct-Gap Semiconductor. Physical Review Letters.

[3] He, K. *et al*. Tightly bound excitons in Monolayer WSe_2_. *Physical Review Letters* **113** (2014). 10.1103/PhysRevLett.113.026803 25062219

[4] Ugeda MM (2014). Giant bandgap renormalization and excitonic effects in a monolayer transition metal dichalcogenide semiconductor. Nature Materials.

[5] Chernikov, A. *et al*. Exciton binding energy and nonhydrogenic rydberg series in Monolayer WS_2_. *Physical Review Letters* **113** (2014). 10.1103/PhysRevLett.113.076802 25170725

[6] Sercombe, D. *et al*. Optical investigation of the natural electron doping in thin MoS_2_ films deposited on dielectric substrates. *Scientific Reports* **3** (2013). 10.1038/srep03489 PMC3860010 24336152

[7] Hanbicki AT (2016). Anomalous temperature-dependent spin-valley polarization in monolayer WS_2_. Scientific reports.

[8] Zhu B, Zeng H, Dai J, Gong Z, Cui X (2014). Anomalously robust valley polarization and valley coherence in bilayer WS_2_. Proceedings of the National Academy of Sciences.

[9] Ross JS (2013). Electrical control of neutral and charged excitons in a monolayer semiconductor. Nature Communications.

[10] Jones AM (2013). Optical generation of excitonic valley coherence in monolayer WSe_2_. Nature Nanotechnology.

[11] Moody G (2015). Intrinsic homogeneous linewidth and broadening mechanisms of excitons in monolayer transition metal dichalcogenides. Nature Communications.

[12] Jakubczyk T (2016). Radiatively limited dephasing and exciton dynamics in MoSe_2_ monolayers revealed with four-wave mixing microscopy. Nano Letters.

[13] Dey, P. *et al*. Optical coherence in atomic-monolayer transition-metal dichalcogenides limited by electron-phonon interactions. *Physical Review Letters* **116** (2016). 10.1103/PhysRevLett.116.127402 27058100

[14] Selig M (2016). Excitonic linewidth and coherence lifetime in monolayer transition metal dichalcogenides. Nature Communications.

[15] Amani M (2015). Near-unity photoluminescence quantum yield in MoS_2_. Science.

[16] Amani M (2016). Recombination kinetics and effects of superacid treatment in sulfur-and selenium-based transition metal dichalcogenides. Nano letters.

[17] Cadiz F (2016). Well separated trion and neutral excitons on superacid treated MoS_2_ monolayers. Applied Physics Letters.

[18] Cadiz F (2016). Ultra-low power threshold for laser induced changes in optical properties of 2d molybdenum dichalcogenides. 2D Materials.

[19] Mak KF (2012). Tightly bound trions in monolayer MoS_2_. Nature Materials.

[20] Komsa, H.-P. *et al*. Two-dimensional transition metal dichalcogenides under electron irradiation: Defect production and doping. *Physical Review Letters* **109** (2012). 10.1103/PhysRevLett.109.035503 22861869

[21] Hong J (2015). Exploring atomic defects in molybdenum disulphide monolayers. Nature Communications.

[22] Britnell L (2012). Electron tunneling through ultrathin boron nitride crystalline barriers. Nano letters.

[23] Tongay S (2013). Broad-range modulation of light emission in two-dimensional semiconductors by molecular physisorption gating. Nano letters.

[24] Miller B, Parzinger E, Vernickel A, Holleitner AW, Wurstbauer U (2015). Photogating of mono-and few-layer MoS_2_. Applied Physics Letters.

[25] Kylänpää, I. & Komsa, H.-P. Binding energies of exciton complexes in transition metal dichalcogenide monolayers and effect of dielectric environment. *Phys*. *Rev*. *B* **92** (2015).

[26] Wang G (2015). Polarization and time-resolved photoluminescence spectroscopy of excitons in MoSe_2_ monolayers. Applied Physics Letters.

[27] Lin Y (2014). Dielectric screening of excitons and trions in single-layer MoS_2_. Nano letters.

[28] Malitson I (1965). Interspecimen comparison of the refractive index of fused silica. Josa.

[29] Gorbachev RV (2011). Hunting for monolayer boron nitride: optical and raman signatures. Small.

[30] Knorr A, Hughes S, Stroucken T, Koch SW (1996). Theory of ultrafast spatio-temporal dynamics in semiconductor heterostructures. Chemical physics.

[31] Korn T, Heydrich S, Hirmer M, Schmutzler J, Schüller C (2011). Low-temperature photocarrier dynamics in monolayer MoS_2_. Applied Physics Letters.

[32] Cadiz, F. *et al*. Excitonic linewidth approaching the homogeneous limit in MoS_2_ based van der waals heterostructures: accessing spin-valley dynamics. *Phys. Rev. X**7*, 021026 (2017).

[33] Ajayi, O. *et al*. Approaching the intrinsic photoluminescence linewidth in transition metal dichalcogenide monolayers. *2D Mater.* **4** 031011 (2017).

[34] Castellanos-Gomez A (2014). Deterministic transfer of two-dimensional materials by all-dry viscoelastic stamping. 2D Materials.

