# Supplementary material for: Direct exciton emission from atomically thin transition metal dichalcogenide heterostructures near the lifetime limit
Source: Sci Rep. 2017 Sep 28;7:12383. doi: 10.1038/s41598-017-09739-4 (PMC5620059; doi:10.1038/s41598-017-09739-4)
Supplement: Supplementary file 1 — Supplementary information [file 41598_2017_9739_MOESM1_ESM.pdf]

Supplementary information: Direct exciton emission from atomically thin transition metal dichalcogenide heterostructures near the lifetime limit

Jakob Wierzbowski[1,2,*], Julian Klein[1,2], Florian Sigger[1], Christian Straubinger[1], Malte Kremser[1], Takashi Taniguchi[3], Kenji Watanabe[3], Ursula Wurstbauer[1,2], Alexander W. Holleitner[1,2], Michael Kaniber[1,2], Kai Müller[1], and Jonathan J. Finley[1,2]

[1]Walter Schottky Institut and Physik Department, Technische Universität München, Am Coulombwall 4, 85748 Garching, Germany
[2]Nanosystems Initiative Munich (NIM), Schellingstr. 4, 80799 Munich, Germany
[3]National Institute for Materials Science, Tsukuba, Ibaraki 305-0044, Japan
[*]jakob.wierzbowski@wsi.tum.de

ABSTRACT

Please see main text.

S1 TMDC crystals

The employed n-$MoSe_2$ and p-WSe_2 crystals were purchased at HQGraphene (www.hqgraphene.com), Netherlands and the MoS_2 was purchased at SPI Supplies (www.2spi.com), USA. The doping information is provided on the corresponding websites and is consistent with our measurements on electrically contacted monolayer samples (data not shown).

S2 Effects of annealing

In order to illustrate the influences of annealing, we present in Fig. S1 two microscope and AFM phase images[1] of a region of a $MoSe_2$/hBN structure, before and after the annealing process. We employ the phase images due to contrast reasons. The RMS analysis was carried out on the actual height profiles. Figure S1a shows a $MoSe_2$ monolayer on a (14.0 ± 0.7) nm thick hBN multi-layer crystal. Although hardly visible in the optical microscope image, the $MoSe_2$ layer strongly wrinkles in the upper half part of the flake. The bottom half and the region on the SiO_2 exhibit bubble formation. This contrast can be attributed to different speeds and contact pressures during the viscoelastic stamping process. Figure S1b shows the device structure after an annealing procedure at 150 °C for 20 min. We note significant changes in the monolayer's morphology. To further quantify the roughness, we define regions indicated as solid and dashed squares with an area of $9\,\mu m^2$ on the hBN region and $4\,\mu m^2$ on the SiO_2, respectively, and calculate the RMS roughness for these areas. The RMS roughness on the SiO_2 and hBN significantly changes from $\sigma_{SiO_2} = 2.27$ nm and $\sigma_{hBN} = 2.35$ nm to $\sigma'_{SiO_2} = 1.61$ nm and $\sigma'_{hBN} = 1.02$ nm after annealing. The bubble density reduces while smaller bubbles aggregate in bigger bubbles which we attribute to water and polymer residue from the viscoelastic stamping with PDMS under ambient conditions[2,3]. We note, that the left part of the monolayer is shifted by 2.5 µm to the right compared to the as-exfoliated image. This could be explained by the low vapour pressure of the water residue between the different crystals inside the annealing device. The expanding gas leaving the structure lifts parts of the TMDC, while the $MoSe_2$ minimises its surface energy on the hBN in the process by moving inwards. Overall, we observe a smoother $MoSe_2$ surface on the hBN substrate that is consistent with recent works[3].

S3 Peak and linewidth distributions

In the main text we use combined plots for the peak positions and the linewidths of $MoSe_2$ and WSe_2 in different configurations (Fig. 1d and Fig. 2d). In order to unfold these plots, here we show the individual distributions for both parameters.

S3.1 $MoSe_2$

Figure S2 shows the obtained histograms after fitting of the spectral data for the different $MoSe_2$ configurations discussed in the main text. The left panels of Fig.S2a show the evolution of the peak positions of the neutral exciton X and the negatively charged exciton T. The corresponding linewidth distributions are presented in Fig.S2b.

S3.2 WSe_2

Figure S3 shows the peak positions (a) and linewidths (b) distributions for the WSe_2/SiO_2 and hBN/WSe_2/hBN configurations.

S3.3 Statistics summary

For clarity, we list all obtained statistical relevant values from the main text in table S1.

	Neutral exciton peak energy (meV)	Neutral exciton linewidth (meV)	Trion peak energy (meV)	Trion linewidth (meV)
$MoSe_2/SiO_2$	1663.1 ± 1.2	5.0 ± 0.5	1631.8 ± 1.3	7.0 ± 0.8
$MoSe_2$/hBN	1661.8 ± 2.0	6.3 ± 1.0	1630.3 ± 2.0	8.4 ± 1.3
$MoSe_2$/hBN (annealed)	1651.0 ± 3.9	5.7 ± 1.5	1623.0 ± 3.7	4.8 ± 1.5
hBN/$MoSe_2$/hBN	1652.1 ± 2.3	4.7 ± 0.9	1623.6 ± 2.4	4.9 ± 1.3
WSe_2/SiO_2	1744.8 ± 2.0	10.3 ± 0.7	1710.4 ± 1.6	10.1 ± 2.1
hBN/WSe_2/hBN	1743.8 ± 4.8	9.8 ± 1.4	1710.5 ± 3.8	8.4 ± 1.9
MoS_2/SiO_2	1947.4 ± 0.3	14.7 ± 0.7	1910.7 ± 0.3	23.4 ± 0.8
hBN/MoS2/hBN	1955.8 ± 0.5	4.8 ± 1.0	1926.2 ± 0.5	6.8 ± 0.9

Table S1. Summarised peak energies and line widths of the different material combinations discussed in the main text.

References

1. Horcas, I. *et al.* Wsxm: a software for scanning probe microscopy and a tool for nanotechnology. *Rev. Sci. Instruments* **78**, 013705 (2007).
2. Castellanos-Gomez, A. *et al.* Deterministic transfer of two-dimensional materials by all-dry viscoelastic stamping. *2D Mater.* **1**, 011002 (2014).
3. Khestanova, E., Guinea, F., Fumagalli, L., Geim, A. & Grigorieva, I. Universal shape and pressure inside bubbles appearing in van der waals heterostructures. *Nat. Commun.* **7** (2016).

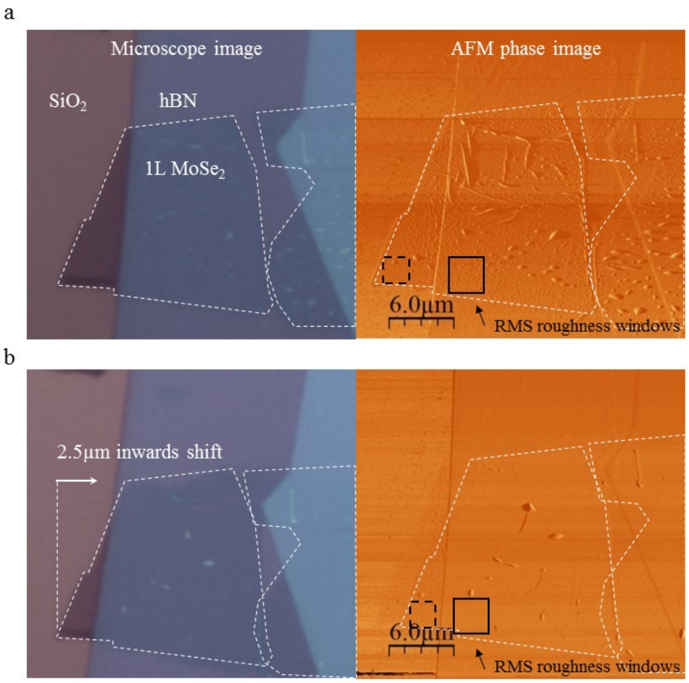

Figure S1. Optical microscope and AFM phase images of $MoSe_2$. Annealing effects of $MoSe_2$ on hBN. (a) Microscope (left panel) and AFM phase images (right panel) of $MoSe_2$ before annealing. The white dashed regions show the $MoSe_2$ monolayer crystal partially covering SiO_2 and hBN regions. The solid (dashed) black $3 \times 3\,\mu m^2$ ($2 \times 2\,\mu m^2$) squares indicates a region for RMS roughness analysis. (b) $MoSe_2$ monolayer after annealing at 150 °C. The solid white arrow indicates a lateral shift of the monolayer material into the hBN region. The phase images are shown to emphasise the morphology. The RMS roughness analysis was performed on the actual height profiles.

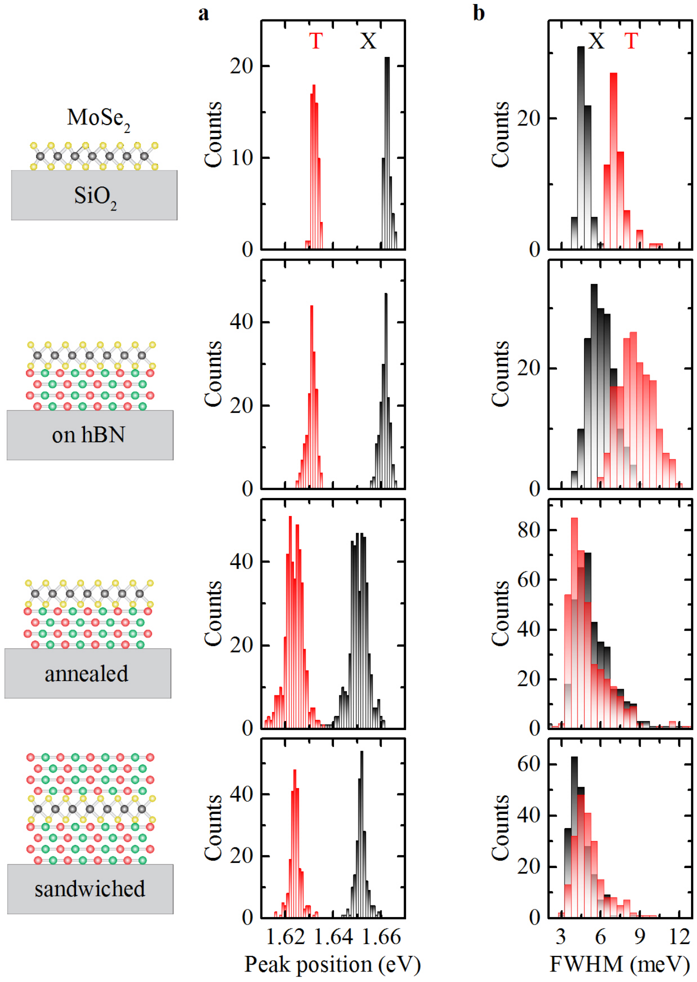

Figure S2. $MoSe_2$ statistics. $MoSe_2$ peak energy and linewidth evolutions for different substrate and capping configurations. (a) Trion T (red) and neutral exciton X (black) peak energy distributions. The upper panel shows the fitted peak positions for $MoSe_2$ on SiO_2. The central panels show the fitted peak positions of both exciton species for the case of $MoSe_2$ directly exfoliated on top of hBN and the positions after an annealing step. The lower panel shows the peak energies for $MoSe_2$ sandwiched between hBN layers. (b) Corresponding linewidth distributions for $MoSe_2$ on SiO_2 (upper panel), $MoSe_2$ on hBN (central panels) and the hBN/$MoSe_2$/hBN heterostructure (lower panel).

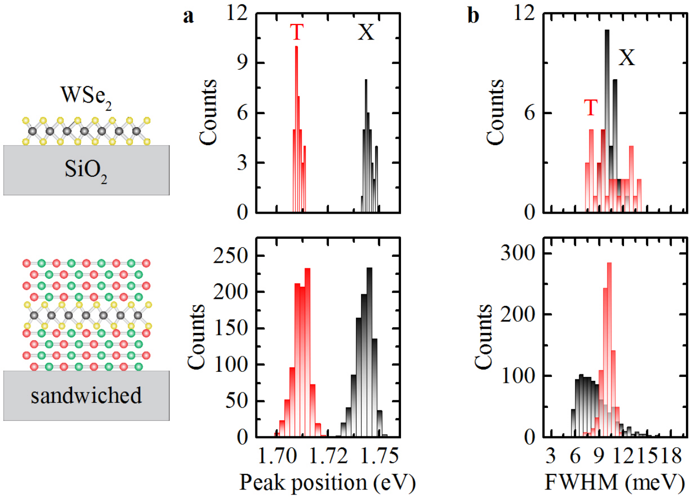

Figure S3. **WSe_2 statistics.** WSe_2 peak energy and linewidth evolutions for different substrate and capping configurations. (a) Trion T (red) and neutral exciton X (black) peak energy distributions. The upper panel shows the fitted peak positions for WSe_2 on SiO_2. The lower panel shows the peak energies for WSe_2 sandwiched between hBN layers. (b) Corresponding linewidth distributions for WSe_2 on SiO_2 (upper panel) and the hBN/WSe_2/hBN heterostructure (lower panel).
